# The Surgical Clinic of the Philadelphia Hospital of Oral Surgery

**Published:** 1881-01

**Authors:** James E. Garretson

**Affiliations:** Philadelphia


ARTICLE VI.

### THE SURGICAL CLINIC OF THE PHILADELPHIA HOSPITAL OF ORAL SURGERY.

SERVICE OF PROF. JAMES E. GARRETSON, M. D., D. D. S., PHILADELPHIA.

Reported by C. H. Browning.

GENTLEMEN:—The first case which I have the pleasure of introducing to the class this morning is that of a private patient, who out of consideration of the opportunity afforded me of demonstrating to you the applicability of the sulphuric acid treatment in various affections of the bones of the face, has kindly consented to appear at our clinic. Before proceeding with the investigation, a few remarks on the general pathology of caries will not be out of place.

As, in preceeding lectures, I have explained that inflammations of whatever character, arise out of the presence of an irritant, and that nature, finding it impossible to relieve herself of its presence through that process which we have studied as resolution, seeks the expulsion of the incumbent by and through the medium of evicunnallation ; a circumscribed condition of the tissues, was familiar to him, which favors the molecular disintegration, liquifaction and expulsion of the tissues involved, and these parts, the agent of offences.

Caries, gentlemen, is a disease of the bones very analogous to ulceration of the soft parts ; a lesion inflammatory in type, the affected parts passing through the successive stages of increased vascularity, softening, molecular disintegration of its mineral and organic constituents with final liquefaction and discharge.

Diagnosis of caries lies in discrimination of the condition from an allied disease affecting the same structures ; namely, necrosis. Apropos, it is scarcely possible to arrive at an absolute differentiation until ulceration and discharge of the superimposed tissues shall have indirectly exposed the part, after which occurrence the diagnosis is quickly cleared up by a judicious use of the probe. Although this, just suggested, is generally true there are signs of great diagnostic value, appreciation of which, may enable the surgeon to conjecture the character of the morbid action before the giving way of structure ; as for example, a lesser amount of pain and constitutional disturbance attendant upon caries, the seat of the morbid phenomena, caries, usually affecting the short bones, while necrosis is more commonly met with in the long. Again, information which he derived from a knowledge of exciting causes, syphilitic asteitis more commonly terminating in necrosis than caries, the reverse being the case when the affection is dependent upon strumous or scorbutic origin.

Returning to our patient, you will notice when looking into his mouth, that he has lost the right lateral incisor tooth which, as I learn from the history of the case, was extracted by his dentist, that when pressure is made upon the opening there exudes a small quantity of pus. Passing a probe into the sinus it comes in contact with something which feels like honey-comb, a touch, which as I have frequently told you, is peculiarly significant of caries. Examining into the previous history of the case, I find the condition here present to have been precedent by all of the phenomena of ordinary alveolar abscess, which phenomena originated, probably, out of the irritation existing in a carious tooth. As to what however, may have been the primary cause of the morbid action the condition is one of caries of the gum. My immediate object in bringing the case before you is to show a condition in which sulphuric acid, as a therapeutic agent, is invaluable.

In all cases of caries in which the disease is strictly localized, in which the process of liquifaction is slow, and where an application can be made with facility, and without endangering adjacent structures, sulphuric acid constitutes the easiest and most effective means of treatment that I have knowledge of. All that is required for the application of the agent is a small piece of absorbent cotton upon which is placed the dilute acid the aromatic being employed, which cotton is passed into the sinus on the end of a probe and allowed to come in direct contact with the exposed bone. A manner of application, for better, however, a manner always adopted in my own practice, consists in making free exposure of the diseased bone and applying the cotton direct and fully saturated. Aromatic sulphuric acid does no harm to the soft parts. To expose carious bone, good practice consists in making a crucial incision through the overlying structure and packing cotton into the wound, which cotton being allowed to remain until the following day swells and exposes the bone most satisfactorily. The part thus made ready is to be dried by means of bibulous paper, and with the patient lying upon the proper side, profuse application of the acid is made by means of the absolving cotton.

In the present instance the patient will lie upon the table until the bone has taken up a free quantity of the acid. Allowed then to get up he will carry the application in its relation with the carious part until the morrow.

Watching the case you will understand how sulphuric acid cures caries of bones. No requisition is made. In a week, or it may not [illegible] or [illegible] or three, red points will show themselves in and amongst the mass. This is the beginning of what is aimed for. A very little time later and the whole surface is sprouting out granulations. Stop here the acid and leave the case to nature.

Owing to its affinity for water and the facility with which it combines with the inorganic salts of the devitalized bone it assists in effectually removing that which has in itself become a source of irritation.

